# Mouse Cardiac Pde1C Is a Direct Transcriptional Target of Pparα

**DOI:** 10.3390/ijms19123704

**Published:** 2018-11-22

**Authors:** Varsha Shete, Ning Liu, Yuzhi Jia, Navin Viswakarma, Janardan K. Reddy, Bayar Thimmapaya

**Affiliations:** 1 Department of Pathology, Feinberg School of Medicine, Northwestern University, Chicago, IL 60611, USA; varsha.shete@gmail.com (V.S.); ning.liu@northwestern.edu (N.L.); y-jia@northwestern.edu (Y.J.); jkreddy@northwestern.edu (J.K.R.); 2 Department of Surgery, Division of Surgical Oncology, University of Illinois at Chicago, Chicago, IL 60612, USA; navinv@uic.edu; 3 Department of Microbiology and Immunology, Feinberg School of Medicine, Northwestern University, Chicago, IL 60611, USA

**Keywords:** PPARα, phosphodiesterase 1C, cAMP, Med1

## Abstract

Phosphodiesterase 1C (PDE1C) is expressed in mammalian heart and regulates cardiac functions by controlling levels of second messenger cyclic AMP and cyclic GMP (cAMP and cGMP, respectively). However, molecular mechanisms of cardiac *Pde1c* regulation are currently unknown. In this study, we demonstrate that treatment of wild type mice and H9c2 myoblasts with Wy-14,643, a potent ligand of nuclear receptor peroxisome-proliferator activated receptor alpha (PPARα), leads to elevated cardiac *Pde1C* mRNA and cardiac PDE1C protein, which correlate with reduced levels of cAMP. Furthermore, using mice lacking either *Pparα* or cardiomyocyte-specific *Med1*, the major subunit of Mediator complex, we show that Wy-14,643-mediated *Pde1C* induction fails to occur in the absence of *Pparα* and *Med1* in the heart. Finally, using chromatin immunoprecipitation assays we demonstrate that PPARα binds to the upstream *Pde1C* promoter sequence on two sites, one of which is a palindrome sequence (agcTAGGttatcttaacctagc) that shows a robust binding. Based on these observations, we conclude that cardiac *Pde1C* is a direct transcriptional target of PPARα and that *Med1* may be required for the PPARα mediated transcriptional activation of cardiac *Pde1C*.

## 1. Introduction

Heart failure is the result of a multitude of malfunctions associated with decreased cardiac output, dilatation, or hypertrophy of the myocardium, loss of contractility, and impaired mitochondrial function. Phosphodiesterases (PDEs) play an important role in regulating cardiac contractile function owing to their ability to modulate levels of the critical second messengers cyclic AMP and cyclic GMP (cAMP and cGMP, respectively) [1]. These cyclic nucleotides control a number of cardiac functions including acute effects on cardiomyocyte contraction and mitochondrial function as well as chronic effects on growth and metabolism [2,3]. Phosphodiesterases constitute an important class of enzymes that includes 11 gene-related families of isozymes *Pde1* to *Pde11*. Out of these, PDE1 family is stimulated by intracellular Ca^2+^/Calmodulin (CaM) [4] and notably, the isoform subtype PDE1C hydrolyzes both cAMP and cGMP with similar high affinity. The *Pde1C* isoform is expressed in mouse, rat, and human heart, with the highest levels of expression in human cardiomyocytes [5]. PDE1C has been shown to modulate cardiomyocyte function, vascular smooth muscle cell proliferation, and migration [6]. Recently it has been shown that activation of cAMP hydrolysis by PDE1C promotes cardiomyocyte apoptosis [7]. Despite multiple evidence of the important role of PDE1C in cardiac functions, the molecular mechanisms that regulate its level and activity remain to be explored.

Peroxisome-proliferator activated receptors (PPARs) belonging to the nuclear receptor superfamily regulate a large number of metabolic processes, including those involved in energy metabolism [8,9,10,11]. PPARs, in particular PPARα, activate the fatty acid oxidation genes by directly binding to the cognate sites on the promoters of these genes. It is expressed at high levels in liver, kidney, brown fat, muscle, and heart, and contributes to fatty acid oxidation in these tissues [11,12]. Members of the PPAR family play vital roles in cardiac functions in that PPARα and PPAR-β/δ were shown to regulate genes involved in cardiac lipid metabolism [13]. We recently noted that mice lacking cardiac-specific expression of Mediator subunit 1 (*Med1*), a key subunit of the mammalian Mediator complex, manifest dilated cardiomyopathy and heart failure [14]. This phenotype was accompanied by severely reduced expressions of several genes including cardiac *Pparα* and *Pde1C* [14]. The mammalian Mediator is a large multiprotein complex that orchestrates tissue specific transcriptional activation [15]. Of interest is that MED1 subunit of this complex interacts with PPARα in order to coordinate downstream functions such as energy dissipation and fatty acid oxidation [16]. Indeed, ablation of *Med1* expression leads to a reduced expression of PPARα regulated genes such as fatty acid oxidation enzymes in liver and heart [14,17].

Balanced fatty acid oxidation and energy metabolism are critical for normal cardiac functions. For example, decreased fatty acid oxidation due to reduced PPARα expression in the heart results in cardiac energy deficiency, which is associated with heart failure [18,19]. On the other hand, mice that overexpress PPARα in the heart display increased fatty acid oxidation and triglyceride accumulation leading to left ventricular hypertrophy and cardiac dysfunction [20,21]. Based on these studies, it is evident that PPARα influences cardiac function owing to its major role in controlling fatty acid oxidation in the heart. However, a role of PPARα in the regulation of cardiac genes other than the ones involved in fatty acid oxidation remains to be explored. Recent studies have shown that chronic activation of PPARα results in impaired contractile function, reduced mitochondrial respiration and increased coupling, and results in decreased ejection fraction and pumping efficiency [22]. However, whether PPARα is directly involved in transcriptional regulation of cardiac genes such as those involved in calcium handling pathway is unclear. In this study, we identify cardiac *Pde1C* as a direct transcriptional target of PPARα and demonstrate that *Med1* may control *Pde1C* expression in the mouse heart via PPARα signaling. To our knowledge, this is the first report of experimentally demonstrating PPARα directly regulating a cardiac gene outside the realm of classical fatty acid metabolism.

## 2. Results

### 2.1. Treatment with PPARα Agonist Leads to Increased Pde1C mRNA Expression in H9c2 Cardiomyocytes

**Figure 1:**
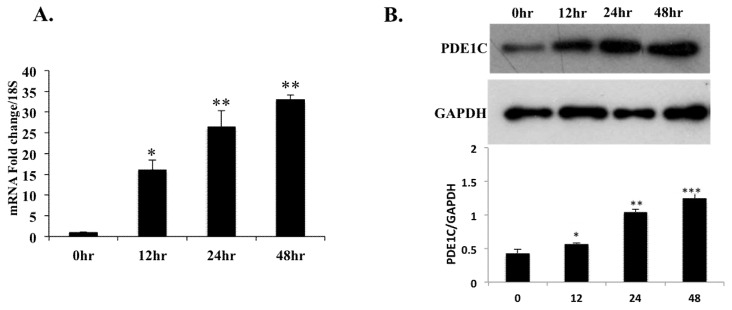
PPARα agonist Wy-14,643 treatment increased *Pde1C* mRNA and PDE1C protein expression in H9c2 cardiomyocytes: H9c2 cardiomyocytes were treated with 100 μM Wy-14,643 for 12-, 24-, and 48-h. Total RNA and total protein from cells were used for RT-qPCR and Western blot analysis, respectively. (**A**) *Pde1C* mRNA levels in Wy-14,643-treated H9c2 cardiomyocytes increased by 16-, 26-, and 33-fold at 12-, 24-, and 48-h, respectively. (**B**) Total protein extracts from H9c2 cells treated with Wy-14,643 for 12-, 24-, and 48-h was analyzed by Western blot using anti-PDE1C antibody, GAPDH was used as loading control. PDE1C protein bands were quantified (lower panel) as described in Section 4 and normalized to GAPDH. PDE1C protein levels increased upon Wy-14,643 treatment. Data was analyzed by *t*-test, *n* = 4–6 per group, * *p* < 0.05, ** *p* < 0.01, *** *p* < 0.001.

To test whether cardiac genes respond to a PPARα agonist in vitro in isolated cells and whether PPARα binds to the *Pde1C* promoter, we used H9c2 rat cardiomyocytes in these assays. H9c2 cells were treated with Wy-14,643, a synthetic ligand specific for PPARα [23,24,25,26] as described in Section 4, and RNA from the treated cells were subjected to RT-qPCR analysis. *Pde1C* mRNA levels increased by 16-, 26-, and 33-fold upon Wy-14,643 treatment at 12-, 24-, and 48-h time points, respectively (Figure 1A).

Protein analysis by Western blot was in agreement with the RT-qPCR analysis. PDE1C protein levels showed continued increase in a time-dependent manner upon Wy-14,643 treatment of H9c2 cardiomyocytes (Figure 1B).

### 2.2. Treatment with PPARα Agonist Wy-14,643 Increased Pde1C mRNA and PDE1C Protein Expression in the Hearts of WT Mice

**Figure 2:**
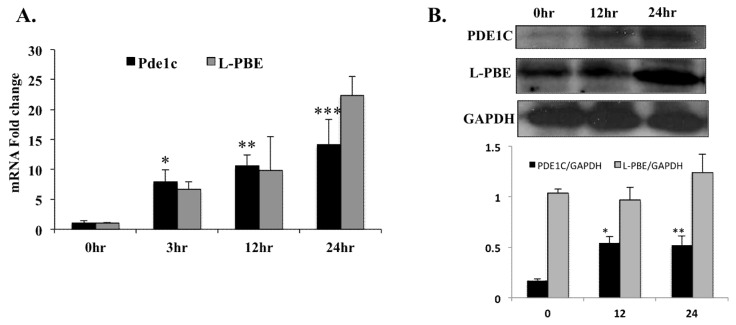
In WT mice, injection with PPARα agonist Wy-14,643 induced *Pde1C* mRNA and PDE1C protein expression in the heart: WT mice were injected with 250 mg/kg body weight of Wy-14,643 via i.p. for 3-, 12-, and 24-h. At each time point mouse hearts were harvested and total RNA as well as total protein from heart lysates were analyzed by RT-qPCR and Western blot, respectively. (**A**) Cardiac *Pde1C* mRNA levels in Wy-14,643-treated mice increase by 8-, 11-, and 14-fold at 3-, 12-, and 24-h, respectively. Similarly, cardiac mRNA levels of positive control *L-pbe* increase upon Wy-14,643 injection of the mice. (**B**) Total protein from hearts was analyzed by Western blot using anti-PDE1C antibody. GAPDH was used as loading control. PDE1C and L-PBE protein bands were quantified as described in Section 4 and normalized to GAPDH levels. PDE1C protein levels increased upon Wy-14,643 treatment. Data was analyzed by *t*-test, *n* = 4–6 per group, * *p* < 0.05, ** *p* < 0.01, *** *p* < 0.001.

To determine the effect of PPARα activation on the *Pde1C* mRNA and PDE1C protein levels in the heart, WT mice were injected with PPARα agonist Wy-14,643 and hearts collected at 3 h, 12 h, and 24 h after the injection. Total mRNA were isolated from the hearts, reverse transcribed then subjected to RT-qPCR. The RT-qPCR analysis showed 8-, 11-, and 14-fold increases in *Pde1C* mRNA levels, at 3-, 12-, and 24-h post injection, respectively (Figure 2A). Enoyl-CoA, hydratase/3-hydroxyacyl CoA dehydrogenase (L-PBE), the bifunctional enzyme in peroxisomal fatty acid oxidation pathway, was used as positive control. *L-pbe* has been shown to be a bonafide Wy-14,643 inducible gene in various tissues [11]. mRNA levels of *L-pbe* in the heart were also induced in a time dependent manner upon Wy-14,643 injection in WT mice, confirming that the agonist was effective (Figure 2A).

PDE1C protein levels in the hearts of mice injected with Wy-14,643 were analyzed by Western blot. PDE1C protein levels increased at 12 h and 24 h after Wy-14,643 treatment in agreement with the increased mRNA levels (Figure 2B). Similarly, protein levels of the positive control L-PBE in the heart also increased in a time dependent manner upon the Wy-14,643 injection in WT mice.

### 2.3. Treatment with PPARα Agonist Wy-14,643 Led to Increased mRNA Levels of Several Members of PDE Family in H9c2 Cardiomyocytes

**Figure 3:**
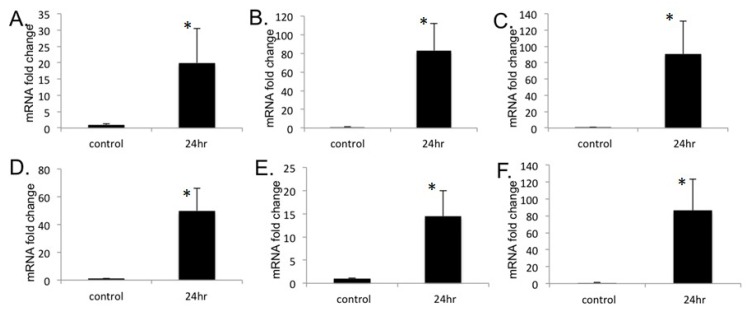
PPARα agonist Wy-14,643 treatment of H9c2 cardiomyoblasts for 24 h induced mRNA expressions of *Pde2*, *Pde6*, *Pde7*, *Pde8*, *Pde10*, and *Pde12*: H9c2 cells were treated with 100 μM Wy-14,643 for 24 h. Total RNA from cells were used for RT-qPCR analysis. (**A**) *Pde2* mRNA levels increased by 20-fold. (**B**) *Pde6* mRNA levels increased by 83-fold. (**C**) *Pde7* mRNA levels increased by 91-fold. (**D**) *Pde8* mRNA levels increased by 50-fold. (**E**) *Pde10* mRNA levels increased by 14-fold. (**F**) *Pde12* mRNA levels increased by 87-fold. GAPDH was used as loading control. Data was analyzed by *t*-test, *n* = 4–6 per group; all sample values were compared to control values. * *p* < 0.001.

The PDE family of enzymes consists of 11 major subtypes and the heart expresses several of them [27,28]. In order to investigate the effects of Wy-14,643 treatment on members of PDE family other than Pde1C, H9c2 cells were treated with Wy-14,643 as described (see Section 4) and RNA from the treated cells were subjected to RT-qPCR analysis. The RT-qPCR analysis showed mRNA levels of Pde2, Pde6, Pde7, Pde8, Pde10, and Pde12 increased by 20-, 83-, 91-, 50-, 14-, and 87-fold upon the treatment with Wy-14,643 as compared with the control (Figure 3).

### 2.4. Wy-14,643 Treatment did not Induce Pde1C mRNA Expression in the Liver of WT Mice

Liver is a metabolically active tissue that expresses significant levels of PPARα, which regulates important biological functions in the liver such as fatty acid oxidation and glucose homeostasis. In order to examine the effects of Wy-14,643 treatment on the mRNA expression of *Pde1C* in the liver, we injected WT mice with a single dose of 250 mg/kg body weight of Wy-14,643 and excised liver and heart tissue 24 h after the treatment. Total RNA was extracted and subjected to RT-qPCR as described in Section 4. *L-pbe* was used as a positive control. As expected, we observed a significant increase in mRNA levels of *L-pbe* in both liver and heart upon Wy-14,643 treatment. However, *Pde1C* mRNA levels in the liver did not change upon Wy-14.643 treatment, as opposed to the heart, where significant increase was observed (9-fold; Table 1).

### 2.5. Cardiac Pde1C mRNA Levels did not Alter upon Wy-14,643 Injection of Pparα^-/-^ and TmcsMed1^-/-^ Mice

We recently showed that mice lacking cardiomyocyte-specific *Med1* expression have reduced *Pparα* expression as well as reduced expressions of important genes involved in PPARα-regulated cardiac energy metabolism, cardiac muscle contraction, and calcium signaling, including *Pde1c* [14]. Furthermore, in that study we showed that tamoxifen-inducible cardiac-specific deletion of *Med1* led to the development of cardiomyopathy and death of mice within four weeks, thus underscoring the involvement of Med1 in regulating important cardiac-specific functions [14].

Since Wy-14,643 is a PPARα-specific agonist, we reasoned that induction of cardiac *Pde1C* upon Wy-14,643 injection in WT mice is a result of activated cardiac PPARα.

To examine the involvement of *Pparα* and *Med1* in the induction of cardiac *Pde1C*, we treated mice lacking systemic *Pparα* expression (*Pparα*^−/−^) and mice with tamoxifen inducible cardiac-specific *Med1* gene deletion (Tmcs*Med1*^−/−^) with PPARα-specific agonist Wy-14,643 and collected hearts after 24 h for further analyses (see Section 4 for details). RT-qPCR analysis showed that in both genotypes, *Pde1C* mRNA levels did not alter upon the treatment with the PPARα agonist, suggesting that increased *Pde1C* expression in WT mice upon Wy-14,643 treatment was dependent on *Pparα* as well as on *Med1* expression (Table 2).

### 2.6. PPARα Binds at Two Sites of the Pde1C Promoter

**Figure 4:**
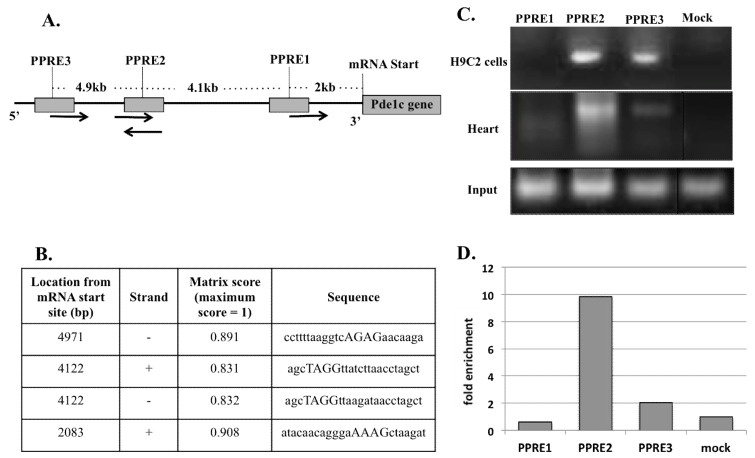
Chromatin immunoprecipitation assay of H9c2 cells and WT mouse heart genomic DNA revealed that PPARα binds to two sites in the 5kb promoter region of *Pde1C* gene. (**A**) Schematic representation of 5kb *Pde1C* promoter region and locations of putative PPRE on the promoter. PPRE2 is a palindrome sequence. The arrows show plus and minus strands of DNA, respectively. (**B**) Specific locations and sequences of putative PPREs on *Pde1C* promoter as analyzed by Genomatix MatInspector software. (**C**) Total lysates from H9c2 cells and mouse heart treated with Wy-14,643 were analyzed by ChIP using PPARα antibody. PPARα bound to PPRE2 and PPRE3 in both H9c2 cells and WT mouse hearts. + and – refer to plus and minus strands of DNA, respectively. (**D**) ChIP of mouse heart was followed by RT-qPCR to quantify the extent of PPARα binding to *Pde1C* promoter. PPRE2 and PPRE3 showed 10- and 2-fold increased binding, respectively, as compared to mock. The input panel shown in Figure 4C belongs to that of heart samples. The input data of H9c2 samples not shown here are similar to that of heart samples.

*Pde1C* promoter analysis, conducted using Genomatrix MatInspector software, revealed 3 putative peroxisome-proliferator activated receptor response elements (PPRE) located at 2083 bp, 4122 bp, and 4971 bp upstream of the mRNA start site (see Section 4 for further details) suggesting a high probability of PPARα binding on these sites (Figure 4A). Out of these, PPRE2 at 4122 bp upstream of *Pde1C* mRNA start site was a palindrome sequence (agcTAGGttatcttaacctagc; Figure 4B).

To determine whether the elevated *Pde1C* mRNA expression in the heart and H9c2 cardiomyocytes were due to a direct transcriptional activation of *Pde1C* by PPARα, we analyzed PPARα binding on *Pde1C* promoter by chromatin immunoprecipitation (ChIP) assays using H9c2 cells and hearts of mice treated with Wy-14,643 (see Section 4 for details).

The upper panel in Figure 4C shows ChIP assay PCR gel of H9c2 cells homogenate. Distinct bands for PPRE2 and PPRE3 sites were present; these bands were absent in PPRE1 and mock infected cell samples indicating a definitive interaction of PPARα with PPRE2 and PPRE3 sites. Also noteworthy is that PPARα bound more strongly with PPRE2 site as compared to PPRE3 site. The ChIP assay of total heart homogenates revealed two of the three putative PPREs (PPRE2 and PPRE3) as true PPARα binding sites (Figure 4C, middle panel). Again, a much stronger PPARα interaction with PPRE2 as compared to PPRE3 was evident. RT-qPCR analysis of the DNA bands shown in Figure 4C (middle panel) revealed that the binding of PPARα at PPRE2 and PPRE3 was 10- and 2-fold stronger in the mouse heart homogenate than that of controls, respectively (Figure 4D). The mice heart ChIP results were confirmed in two independent experiments. Similarly, the results of H9c2 ChIP results were confirmed in three independent experiments. In both cases, results were highly reproducible. The results of representative experiments in each case are presented in Figure 4C,D.

These results demonstrate that PPARα bound to the same two PPREs at 4.1 kb and 4.9 kb upstream of the mRNA start site on pde1C promoter, with PPRE2 at 4122 bp showing a much higher binding affinity for PPARα as compared to the PPRE3 site at 2083 bp.

### 2.7. Treatment with PPARα Agonist Wy-14,643 Led to Reduced Cyclic AMP Levels in Mouse Hearts and H9c2 Cardiomyocytes

**Figure 5:**
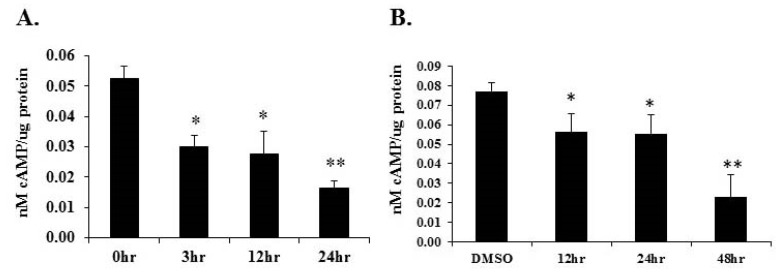
Cyclic AMP (cAMP) protein levels reduced upon Wy-14,643 treatment in H9c2 cardiomyocytes and WT mouse hearts: (**A**) WT mice were injected with 250 mg/kg body weight of Wy-14,643 and hearts were excised at 3-, 12- and 24-h post treatment. cAMP levels in total heart lysates were measured by ELISA and normalized to total protein. (**B**) H9c2 cardiomyocytes were treated with 100 μM Wy-14,643 dissolved in DMSO for 12-, 24-, and 48-h. cAMP levels in total cell extracts were determined by ELISA and normalized to total protein. Samples were compared to controls by *t*-test, *n* = 4–6 per group, * *p* < 0.05, ** *p* < 0.001.

Cyclic AMP has long been established as a crucial modulator of cardiac genes responsible for maintaining normal function. PDE1C controls cyclic AMP levels by hydrolyzing it to 5’-AMP [2,3,5]. To determine the effect of *Pde1C* induction by Wy-14,643 on the cAMP levels, we measured cAMP levels in the hearts of WT mice injected with Wy-14,643 and H9c2 cardiomyocytes treated with Wy-14,643 treatment by using ELISA (see Section 4 for further details). We observed that in heart homogenates, cyclic AMP levels reduced by 43, 47, and 68% at 3-, 12- and 24-h post Wy-14,643 treatment, as measured by ELISA. In H9c2 lysates, cAMP levels reduced by 27, 28, and 70% at 12-, 24-, and 48-h post treatment, respectively (Figure 5A,B). Overall, these results suggest that increased expression of *Pde1C* in the heart correlates with reduced cyclic AMP levels in the WT mouse heart as well as in H9c2 cardiomyocytes.

### 2.8. Troponin I, an Indicator of Failing Heart, is Increased in WT Mouse Hearts upon Wy-14,643 Treatment

**Figure 6:**
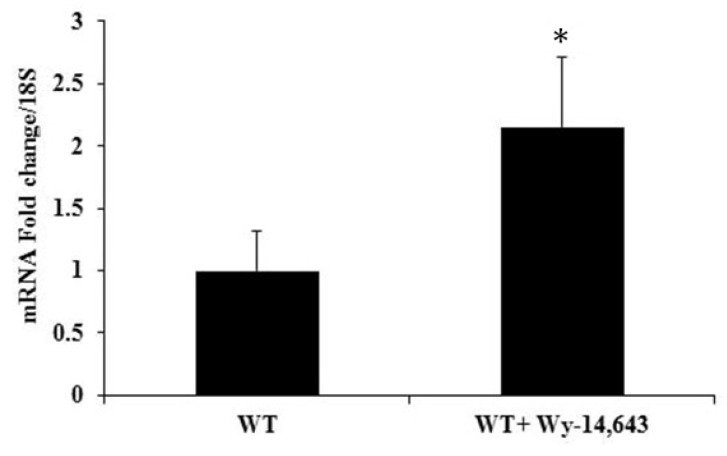
mRNA levels of *cTnI*, an indicator of failing heart, were increased in the hearts of WT mice treated with Wy-14,643: WT mice were injected with 250 mg/kg body weight of Wy-14,643 and hearts were excised 24 h after the treatment. Total RNA from the hearts were analyzed by RT-qPCR, which revealed a ~2-fold increase in mRNA levels of c*TnI* in the hearts of the mice treated with Wy-14,643 as compared to the controls. The data were analyzed by *t*-test, *n* = 4–6 per group, * *p* < 0.05.

To evaluate the effects of *Pde1C* upregulation and reduced cyclic AMP levels on the heart, we analyzed mRNA levels of cardiac troponin I (*cTnI*), a major transcript in the failing heart. Upon Wy-14,643 treatment of WT mice for 24 h, mRNA levels of *cTnI* were increased 2-fold (Figure 6). These results suggest that when cardiac *Pde1C* mRNA and PDE1C protein levels increased in the heart upon Wy-14,643 treatment, they were accompanied by reduced cAMP levels and increased levels of cTNI, indicating a possible onset of cardiac failure.

## 3. Discussion

Peroxisome proliferator-activated receptors (PPARs), a subfamily of nuclear receptor super family, are ligand-activated transcription factors that regulate a large number of physiological processes [9]. Out of the three main PPAR isoforms, PPARα is critical for cellular fatty acid uptake, transport, and oxidation [29]. Previous studies from our laboratory have established that PPARα regulates mitochondrial and peroxisomal fatty acid oxidation in the liver. In those studies, we demonstrated that activation of PPARα by natural or synthetic agonists led to increased expressions and activities of fatty acid oxidation enzymes in the liver leading to higher rates of energy consumption [11]. We also showed that PPARα binds to one or more peroxisome proliferator response elements (PPREs), a DNA consensus sequence consisting of direct repeat of six nucleotides separated by one spacer nucleotide (AGGTCA n AGGTCA), on the promoters of genes encoding fatty acid oxidation genes and regulates their transcription [30]. Owing to its role in energy metabolism, PPARα is highly expressed in tissues with high rates of fatty acid oxidation including the heart [11]. However, it is not known whether PPARα regulates cardiac specific genes, for example, genes involved in the calcium-channeling pathway.

In the present study, we demonstrate that PPARα activation in the heart increases the expression of cardiac *Pde1C*. The enzyme PDE1C is highly expressed in human myocardium and human cardiomyocytes [5]. PDE1C has been shown to regulate vascular smooth muscle cell proliferation and migration, and cardiomyocyte dysfunction. Additionally, *Pde1C* is induced in proliferative human arterial smooth muscle cells [5,31,32,33,34,35]. Moreover, in tissues, PDE1C can degrade cyclic AMP, which conducts several subcellular functions that result in positive inotropic and lusitropic effects [3]. Recent studies have demonstrated that PDE1C is an important regulator of cardiac cAMP signaling and has a direct role in cardiomyocyte death/apoptosis [7,36]. Emerging studies suggest that it may be an important candidate for therapeutic targeting in heart failure [7,37]. Thus, molecular mechanisms that regulate PDE1C in the heart need to be clearly understood in order to evaluate therapeutic potential of this phosphodiesterase.

Wy-14,643 has been widely used as a high-potency and specific PPARα agonist in our laboratory as well as others [23,24,25,26]. Our results clearly demonstrate that treatment with PPARα agonist Wy-14,643 leads to elevated *Pde1C* mRNA and PDE1C protein expressions in H9c2 myoblasts and WT mouse heart (Figure 1 and Figure 2). In addition, we did not observe such treatment effects in *Pparα*^−/−^ mice, suggesting that the increase in cardiac *Pde1C* mRNA levels upon Wy-14,643 treatment depended on *Pparα* (Table 2). In addition, we did not see increase in *Pde1C* mRNA levels in the heart upon Wy-14,643 treatment of Tmcs*Med1*^−/−^ mice, suggesting that *Med1* may be involved in the regulation of cardiac *Pde1C* through PPARα signaling. Moreover, we did not find any change in *Pde1C* mRNA levels in the livers of WT mice treated with Wy-14,643, indicating that increased *Pde1C* mRNA levels upon PPARα activation were observed in the heart but not in the liver (Table 1).

We also showed that increased levels of cardiac PDE1C correlated with reduced cAMP protein levels and increased mRNA levels of cardiac *cTnI*, an indicator of early onset of heart failure. Finally, we demonstrated that PPARα directly binds at two sites on the 5kb upstream promoter region of the *Pde1C* gene, one of which was a palindrome sequences that showed a significantly stronger binding as compared to two other putative binding sites (Figure 4). Thus, we conclude that *Pde1C* is a direct transcriptional target of nuclear receptor PPARα and propose that *Med1* regulates cardiac *Pde1C* via *Pparα* signaling. To our knowledge this is the first report that demonstrates PPARα-mediated regulation of *Pde1C* in the heart.

In our study, promoter analysis of other members of PDE family of enzymes [27,28] also showed potential PPARα binding sites. Further, we also observed significantly increased mRNA expressions of other important members of PDE family, such as *Pde2*, *Pde6*, *Pde8*, *Pde7*, *Pde10*, and *Pde12* upon treatment of H9c2 cells with Wy-14,643 for a period of 24 h. As stated above, PDE1C plays an important role in cardiomyocyte death/apoptosis by regulating cardiac cAMP. Since other forms of the *Pde* family are also induced by Wy-14,643 in heart, it is likely that these isoforms may also contribute to perturbation of cAMP signaling and *cTnI* induction [27]. Clearly, further experiments are warranted to investigate whether increase in mRNA levels are accompanied by elevated protein expressions. If such increase is observed, it will also be of further interest to evaluate whether the increased expressions of other members of PDE family are PPARα -dependent and what role they may play in heart function.

Synthetic PPARα ligands have been implicated in the treatment of heart failure and metabolic diseases such as hyperlipidemia and atherosclerosis due to their ability to stimulate catabolism of lipids, decrease in circulating triglycerides and to favorably alter lipoprotein metabolism [38]. However, PPARα activation has not always been demonstrated to be beneficial in heart function, especially in disease states. For example, in mouse models of pressure-overload cardiac hypertrophy, reactivation of PPARα by ligands resulted in decreased contractile function of hypertrophied heart. This led to the conclusion that PPARα downregulation is essential for maintaining contractility of hypertrophied heart [39]. Moreover, in diabetic states where heart uses fatty acid oxidation as the primary source of energy due to impaired glucose utilization, increased myocardial lipid uptake and accumulation by activated PPAR signaling led to lipotoxic cardiomyopathy [40,41]. Further, it has been shown that in male Wistar rats, Wy-14,643 treatment led to deleterious effects on fatty acid composition in myocardial phospholipids [42]. Overall, studies have shown that ligand mediated PPARα activation can have both beneficial as well as deleterious effects on cardiac functions.

In the present study, we did not observe worsening of ejection fraction or cardiac failure due to treatment of mice with Wy-14,654. Rather, we observed very high levels of mRNA and protein expression of fatty acid oxidation enzymes in the hearts of these mice after Wy-14,643 treatment, as expected.

Our data specifically demonstrates that mouse cardiac *Pde1C* is a direct transcriptional target of PPARα. However, although unlikely, we cannot rule out the formal possibility that the binding is fortuitous and that there may be other targets of PPARα that may be responsible for the activation of *Pde1C.* Our study also raises the possibility that other naturally occurring PPARα agonists such as long-chain unsaturated fatty acids, branched, conjugated, and oxidized fatty acids, and eicosanoids have a potential to induce *Pde1C* expression in the heart. However, further studies are required to evaluate the effect of naturally occurring PPARα agonists on cardiac PDE1C.

It is well known that PPARα activation by ligand binding causes conformational changes in the receptor leading to recruitment of coactivator complexes to facilitate transcription of target genes [43]. Our previous studies have shown that activation of PPARα in the absence of MED1 fails to stimulate downstream functions such as fatty acid oxidation and liver cell proliferation [17]. Lack of MED1 in hepatocytes in vivo mimics the absence of PPARα effects, suggesting that both PPARα and MED1 act in conjunction to facilitate transcription of PPARα -regulated genes in the tissues [17]. Recently, we demonstrated that cardiomyocyte-specific deletion of *Med1* in mice during late gestation, early postnatal development as well as in the adult mice using inducible cardiac *Med1*-deletion resulted in cardiomyopathy-related dilatation and heart failure. In these mice, loss of *Med1* resulted in downregulation of numerous genes involved in distinct cardiac pathways [14]. In our current study, we show that mouse hearts with abolished *med1* expression are similar to the ones lacking *Pparα* in the sense that both fail to show PPARα-ligand induced *Pde1C* expression, which was evident in WT mice.

## 4. Materials and Methods

### 4.1. Mice

Mice with germline deletion of PPARα (*Pparα*^−/−^), tamoxifen inducible heart-specific med1 knockouts (Tmcs*Med1*^−/−^), and wild type littermate control mice used in this study have been described previously [14]. To generate Tmcs*Med1*^−/−^ mice, mice with a loxP flanked allele targeting exons 8–10 of Med1 (*Med1*^fl/fl^ mice) were crossed with Myh6-MCM (tamoxifen-inducible heart specific Cre) transgenic mice purchased from the Jackson Laboratory (Bar Harbor, ME, USA). We have previously shown by RT-qPCR analysis of RNA that *Med1* expression in the heart begins to decrease after 3 days of daily tamoxifen injection and becomes undetectable after 5 days of injection [14]. We thus injected seven-week-old Tmcs*Med1*^−/−^ mice with a daily dose of 65 mg/kg body weight tamoxifen intraperitoneally for 5 days. These mice were then used for treatment with Wy-14,643 to mimic activation of *Pparα* in the absence of *Med1* in the heart.

Mice from all 3 groups (WT, *Pparα*^−/−^ and Tmcs*Med1*^−/−^) were injected intraperitoneally with a single dose of either Wy-14,643 (Gift from Dr. Reddy’s Laboratory, Hyderabad, India), a *Pparα*-specific agonist (250 mg/kg body weight) dissolved in corn oil, or vehicle alone (corn oil). WT mice were euthanized at 3, 12, and 24 h after injection whereas *Pparα*^−/−^ and Tmcs*Med1*^−/−^ mice were euthanized at 24 h after the injection. Animals received food and water ad libitum and lighting was maintained on a 12 h light–dark cycle. Mice were euthanized by intraperitoneal pentobarbital injection at the dose of 150 mg/kg body weight to minimize suffering. All experimental procedures were performed in accordance with the National Institutes of Health guideline for care and use of laboratory animals. All animal studies were reviewed and approved by the Institutional Animal Care and Use Committee Northwestern University (protocol number 2013–3198, 1 July 2013).

### 4.2. Cardiomyoblast Cell Culture

The H9c2 cardiomyoblast cells derived from rat (ATCC CRL1446) were grown at a density of 10^5^ cells/cm^2^ and cultured as monolayers in Dulbecco’s Modified Eagle’s Medium supplemented with 10% (vol/vol) fetal calf serum and 1% (vol/vol) penicillin–streptomycin under an atmosphere of 5% CO_2_ at 37 °C. The culture medium was replaced by fresh medium every 2 days. For treatment group, Wy-14,643 dissolved in DMSO was directly added to the media to a final concentration of 100 μM. Control cells were treated with DMSO alone. Cells were harvested 48 h after the treatment.

### 4.3. Quantitative Real-Time PCR

Total RNA was isolated from the mouse heart and H9c2 cardiomyocytes with TRIzol^®^ as per manufacturer’s instructions (Lifetechnology, Carlsbad, CA, USA). 1 μg of total RNA was reverse transcribed to cDNA using SuperScript™ III First-Strand Synthesis System (Invitrogen, Carlsbad, CA, USA). Three-hundred nanomolar of each specific primer together with 10 to 50 ng of cDNA equivalent with the total RNA was used with SYBR Green (Lifetechnology, Carlsbad, CA, USA) and subjected to RT-qPCR by using an ABI 7300 (Thermo Fisher Scientific, Waltham, MA, USA). All samples were run in triplicates. 18S ribosomal RNA was used to normalize each sample. From the instrument, cycle thresholds (Ct) were obtained for each sample for each gene of interest. To determine changes in gene expression, the ∆∆Ct method was used. The expression of each gene relative to the calibrator was calculated using 2(−∆∆Ct).

### 4.4. Protein Expression Analysis

Total protein from heart and H9c2 cardiomyocytes was subjected to SDS-PAGE and transferred to a nitrocellulose membrane (Invitrogen, Carlsbad, CA, USA). Immunoblotting was performed using anti-PDE1C or anti-LPBE antibodies at a dilution of 1:300 as per the manufacturer’s instructions (Abcam, Cambridge, MA USA). GAPDH was used as loading control (antibody source: Novus biologicals, Centennial, CO, USA). The protein bands were developed with an enhanced chemiluminescence substrate (Proto Glow ECL, National Diagnostics, Atlanta, GA, USA) and were visualized using BioRad ChemiDoc imaging system (Hercules, CA, USA). Protein bands in the blots were quantified using ImageJ software (NIH, Bethesda, MD, USA).

### 4.5. Pde1C Promoter Analysis

To analyze *Pde1C* gene promoter, 5 kb upstream of mRNA start site was analyzed using Genomatix MatInspector software (Genomatix Inc. Ann Arbor, MI, USA) (NCBI gene ID: 18575, Isoform: XM_006505730, Chromosome # 6: NC_000072.6).

### 4.6. Chromatin Immunoprecipitation

Hearts from mice treated with Wy-14,643 (250 mg/kg body weight) for 24 h and H9c2 cells treated with Wy-14,643 (100 μM) for 48 h were used for chromatin immunoprecipitation (ChIP) assay using ab500 ChIP kit (Abcam, Cambridge, MA USA). Thirty milligrams of chopped heart tissue and 1 × 10^6^ H9c2 cells were fixed with 1% formaldehyde and processed according to manufacturer’s protocol. Briefly, fixed tissue or cells were lysed and sonicated using Fisher 300 Sonic Dismembrator (Fisher Scientific, Hampton, NH, USA) to obtain optimal DNA fragments ranging from 200 to 1000 bp. Chromatin was immunoprecipitated with PPARα antibody (ab2779, Abcam, Cambridge, MA USA). or mock IgG followed by reverse crosslinking and DNA purification. PCR was conducted using primers spanning each of the three PPREs on *Pde1C* promoter (Table S1). The PCR product after 35 cycles was run on 1% agarose gel. To quantify the promoter enrichment, 1 μL of purified DNA was analyzed by RT-qPCR, using similar conditions used for PCR. Triplicate C_t_ numbers from sample and mock were used to determine fold enrichment of promoter fragments as compared to mock.

### 4.7. Cyclic AMP Measurement

Cyclic AMP levels in the mouse heart and H9c2 cells were measured by using Cyclic AMP XP^®^ kit (Cell Signaling Technology, Danvers, MA, USA) according to manufacturer’s protocol. Briefly, homogenates from hearts and H9c2 cells were prepared in a lysis buffer and incubated on antibody-coated plates. Absorbance at 450 nm was measured and compared against cAMP standard curve to calculate amount of cAMP in each sample. cAMP levels were expressed as units per mg of protein.

### 4.8. Statistical Analysis

Student’s *t*-test was used to determine whether a sample was significantly different from the control. Differences were considered statistically significant at *p* < 0.05, while *p* < 0.01 represented more significant.

## 5. Conclusions

In the current study, we identified cardiac *Pde1C* as a direct transcriptional target of PPARα in mice. Moreover, we demonstrated that activation of PPARα by specific agonist Wy-14,643 induced cardiac *Pde1C* expression and it was accompanied by reduced cAMP levels in the heart. Overall, our data demonstrated for the first time that PPARα can directly regulate cardiac *Pde1C* expression, a gene outside the realm of classical fatty acid metabolism genes targeted by PPARα and that this regulation may occur through MED1 subunit of the Mediator complex.

## Figures and Tables

**Table 1:** *Pde1C* mRNA levels increase only in the hearts and not in the livers upon Wy-14,643 treatment of WT mice.

	WT LIVER			WT HEART		
	Wy-14,643/Control	*p*-Value		Wy-14,643/Control	*p*-Value	
***L-pbe***	20.07	<0.001	**	378.08	<0.001	**
***Pde1C***	1.05	0.9	NS	9.51	<0.001	**

WT mice were injected with a single dose of 250 mg/kg body weight of Wy-14,643 or vehicle via i.p. Total RNA from hearts and livers were analyzed by RT-qPCR. *Pde1C* mRNA levels increased by ~10-fold in WT hearts; such an increase was not observed in livers of these mice. *L-pbe* was used as a positive control. Data was analyzed by *t*-test, ** *p* < 0.001; NS, not significant.

**Table 2:** Increase in *Pde1C* mRNA levels in WT hearts upon Wy-14,643 treatment is dependent on *PPARα* and *Med1.*

	WT HEART			*Pparα*^−/−^HEART			Tmcs*Med1*^−/−^HEART		
	Wy-14,643/Control	*p*-Value		Wy-14,643/Control	*p*-Value		Wy-14,643/Control	*p*-Value	
***L-pbe***	378.08	<0.001	**	1.2	0.41	NS	0.95	0.06	NS
***Pde1C***	9.51	<0.001	**	0.91	0.38	NS	0.94	0.7	NS

WT, *Pparα*^−/−^ and Tmcs*Med1*^−/−^ mice were injected with a single dose of 250 mg/kg body weight of Wy-14,643 dissolved in corn oil or vehicle via i.p. Total RNA from hearts were analyzed by RT-qPCR, *Pde1C* mRNA levels increased by ~10-fold in WT mouse hearts; no such increase was observed in *Pparα*^−/−^ and Tmcs*Med1*^−/−^ mice. *L-pbe* was used as a positive control and as expected, mRNA levels of *L-pbe* increased significantly only in WT mice upon Wy-14,643 treatment. Data was analyzed by *t*-test, ** *p* < 0.001; NS, not significant.

## Supplementary Materials

Supplementary Materials can be found at http://www.mdpi.com/1422-0067/19/12/3704/s1.
