# Supplementary material for: Mouse Cardiac Pde1C Is a Direct Transcriptional Target of Pparα
Source: Int J Mol Sci. 2018 Nov 22;19(12):3704. doi: 10.3390/ijms19123704 (PMC6321386; doi:10.3390/ijms19123704)
Supplement: Supplementary file 1 [file ijms-19-03704-s001.pdf]

Table S1. Sequences of primers used for ChIP-PCR. Each primer spans PPREs on 5kb *Pde1C* promoter.

	Location from mRNA start site (bp)	Primer Sequence
PPRE1	4971	F: 5′- GAGTCATGCCAGCTAGTGCT -3′
		R: 5′ - AGAGGCCCATTGGACACGCA -3′
PPRE2	4122	F: 5′- GTCTCGAACCTCCCCTCCCC -3′
		R: 5′ - GACATGATGGGTGCACCTGG -3′
PPRE3	2083	F: 5′- CCCTGTGATATTCCCAGTCT -3′
		R: 5′- TGACTTGTGTTTAACTCTCC -3′
